# A Quantitative Analysis of Pulsed Signals Emitted by Wild Bottlenose Dolphins

**DOI:** 10.1371/journal.pone.0157781

**Published:** 2016-07-06

**Authors:** Ana Rita Luís, Miguel N. Couchinho, Manuel E. dos Santos

**Affiliations:** 1 MARE–Marine and Environmental Sciences Centre, ISPA–Instituto Universitário, Lisboa, Portugal; 2 Projecto Delfim–Centro Português de Estudo dos Mamíferos Marinhos, Lisboa, Portugal; University of South Florida, UNITED STATES

## Abstract

Common bottlenose dolphins (*Tursiops truncatus*), produce a wide variety of vocal emissions for communication and echolocation, of which the pulsed repertoire has been the most difficult to categorize. Packets of high repetition, broadband pulses are still largely reported under a general designation of burst-pulses, and traditional attempts to classify these emissions rely mainly in their aural characteristics and in graphical aspects of spectrograms. Here, we present a quantitative analysis of pulsed signals emitted by wild bottlenose dolphins, in the Sado estuary, Portugal (2011–2014), and test the reliability of a traditional classification approach. Acoustic parameters (minimum frequency, maximum frequency, peak frequency, duration, repetition rate and inter-click-interval) were extracted from 930 pulsed signals, previously categorized using a traditional approach. Discriminant function analysis revealed a high reliability of the traditional classification approach (93.5% of pulsed signals were consistently assigned to their aurally based categories). According to the discriminant function analysis (Wilk’s Λ = 0.11, F_3, 2.41_ = 282.75, P < 0.001), repetition rate is the feature that best enables the discrimination of different pulsed signals (structure coefficient = 0.98). Classification using hierarchical cluster analysis led to a similar categorization pattern: two main signal types with distinct magnitudes of repetition rate were clustered into five groups. The pulsed signals, here described, present significant differences in their time-frequency features, especially repetition rate (P < 0.001), inter-click-interval (P < 0.001) and duration (P < 0.001). We document the occurrence of a distinct signal type–short burst-pulses, and highlight the existence of a diverse repertoire of pulsed vocalizations emitted in graded sequences. The use of quantitative analysis of pulsed signals is essential to improve classifications and to better assess the contexts of emission, geographic variation and the functional significance of pulsed signals.

## Introduction

Common bottlenose dolphins, *Tursiops truncatus*, have a complex acoustic repertoire [1–4] comprised of three major types of signals: (i) tonal, omnidirectional, frequency-modulated whistles used as cohesion calls and communication signals [5–7], (ii) highly directional echolocation clicks used in biosonar tasks [8], and (iii) a variety of other pulsed signals, with high repetition rate (above 300 pulses per second) and short inter-click-intervals (less than 3 ms), the “burst-pulses” [9–11].

Although burst-pulses are formed by broadband pulses often similar to echolocation signals, researchers studying acoustic production in delphinids consider these two separate categories. The distinction is supported by the notion that signals with very short inter-click-intervals (ICI) cannot be perceived individually, thus packet emission of clicks may prevent functional sonar [8,11]. However, this simplistic segregation may not reflect the true nature of these signals as the pulses within these high repetition, pulsed signals can be produced with a range of time-frequency parameters [4]. During echolocation tasks, click trains are usually emitted with ICIs longer than the two-way transit time (i.e. required time for a click to propagate to the target, echo back and be processed) [8]. Strikingly, in captivity experiments, it has been verified that bottlenose dolphins are able to adjust their click emission and use burst-pulses with ICIs lower than the two-way transit time during long-range biosonar tasks [12].

Understanding the functional significance of such signals is still challenging, especially since burst-pulses are not yet fully described. Despite the frequent occurrence of burst-pulses both in wild and captive bottlenose dolphins [2,13], the vast majority of dolphin acoustic repertoire studies still focus on whistles and/or echolocation clicks. While the acoustic parameters, context of emissions and functional aspects of whistles and echolocation signals are well documented [14–18], research on burst-pulses is still scarce.

Classical attempts to classify these pulsed emissions rely mainly in their aural characteristics and in graphical aspects of spectrograms, which allows for much subjectivity. As a result, various terminologies, usually onomatopoeic, have been used to report burst-pulses that occur in various aroused behavioural contexts [2,19]. “Creaks” or “buzzes” have been described as detailed echolocation signals, produced during foraging and feeding events (e.g.,[2,20]). Burst-pulses with very high repetition rates (200–1200 clicks/s), that are often recorded during courtship and agonistic interactions, have been classified as “squawks” and “squeaks” [21–23]. Additionally, there are descriptions of “barks”, “screams”, “cracks”, “pops”, “genital buzzes” and “yelps”, observed during agonistic interactions, fright and alarm situations, and courtship and precopulatory behaviours [2, 24–26]. The rhythmic sequences denominated as “Bray series”, recorded during feeding activities, are also part of the diversity of burst-pulses [27–30].

Although the contribution of qualitative methods to the description of dolphins' acoustic repertoires is recognized, the reach of such subjective classifications is limited. Burst-pulses are mainly ultrasonic signals [23], with inter-click-intervals (ICI) too short to be discernible to the human ear [8]. Minor changes in ICIs may be misinterpreted or perceived differently depending on the observer, which may lead to the creation of artificial groups or incorrect classifications. Also, a signal with lower ICI, within human perception abilities, might be easier to classify using qualitative methods.

The use of graphical aspects of spectrograms in pulsed signals classification can also be problematic, due to the graphical representations of acoustic signals are highly dependent on the selected time-frequency parameters and analysis windows. High-repetition pulsed signals may be represented as horizontal bands that resemble harmonics of a tonal sound depending on the analysis windows settings [31].

Distinct packets of pulses emitted by dolphins are still mostly reported under a general designation of burst-pulses, hindering comparisons among populations and diminishing our ability to document geographic variations in the acoustic repertoire of this species. These difficulties require an application for quantitative analysis to facilitate rigorous classification efforts.

The use of multivariate classification techniques based on time-frequency parameters can be useful to overcome the methodological difficulties in pulsed signals categorization.

In this study, we assess the exactness of traditional classifications of pulsed signals by applying a discriminant function analysis (DFA) and hierarchical cluster analysis to a dataset of signals previously categorized using aural and visual classification.

Additionally, we present a quantitative characterization of broadband pulsed signals emitted by wild bottlenose dolphins that may contribute to clarifying the nature, context of emission and functional significance of these puzzling sounds.

## Methods

### Data Collection

Field recordings were made in the Sado estuary, Portugal, and adjacent coastal waters (approximate location of the Sado mouth: 38° 29’ N, 8° 55’ W). All data were collected from a 8.40 m inboard motor vessel during daylight hours (1000 to 1800), on 42 days from April 2011 to March 2014, with sea state ranging from 0 to 3 Beaufort. Whenever a group of dolphins was visually detected, the research vessel was positioned approximately 500 m ahead of the group’s location, with the engines off, and the hydrophone placed at a depth of 3 m. The distance and relative position of the vocalizing individuals could not be assessed.

All acoustic measurements were carried out using a factory-calibrated recording system: a Cetacean Research Technology hydrophone, model C55 (effective sensitivity of -185 dB RMS re 1V = 1μPa, frequency response: 0.008 to 100 kHz ± 3 dB, polarized by a 9 V battery) connected by a 15 m cable to a Fostex FR-2 digital recorder. A high-pass filter of 100 Hz was chosen to avoid self-noise generated by the recording platform and low-frequency vibrations. One-minute duration recordings were made, with a sampling rate of 192 kHz and 24-bit resolution, recording level of the Fostex at 7.5 and trim level at -26 dB. All recordings were stored on Compact Flash memory cards as time-stamped.wav files. The geographic location of each recording was provided by a Garmin Foretrex 301 portable GPS, and dolphin activities and group composition were registered by two experienced independent observers during the acoustic recordings.

### Acoustic Analyses

Recordings were first inspected by two trained independent observers, aurally and visually, using Adobe Audition CS5.5 (Adobe Systems Inc.) with Hamming windows of 512 points, in order to identify and classify the pulsed signals present in each recording.

Pulsed signals were assigned to one of the following pre-established categories, according with graphical and aural characteristics: “Slow click trains”–discernible click trains; “Creaks”–long burst-pulse (>0.2 sec.), aurally similar to a creaking door; “Squawks”–long burst-pulse (>0.2 sec.), with higher repetition rate than “Creaks”, reminiscent of a crying baby; “Short Burst-Pulses (S-BP)”—short burst-pulse (<0.2 sec.), aurally similar to a buzzing bee but brief. Signals that were part of complex vocal sequences—Bray series [26–28] were not included in these analyses due to their specific rhythmic characteristics, and will be discussed in a separate study.

All the identified sounds were rated based on signal-to-noise ratio (SNR) as follows: (i) poor–signal faint and hardly visible on the spectrogram, (ii) fair–signal visible and with a clear start/end on the spectrogram, (iii) good–signal well marked and with a clear start/end on the spectrogram. Non-overlapping signals rated as fair or good where selected for further analysis.

Raven Pro 1.4 (Cornell Lab of Ornithology) with Hamming windows of 512 points, frequency resolution of 93.8 Hz, 50% overlap was used to measure the acoustic parameters of the selected pulsed signals: minimum frequency, maximum frequency, frequency range, peak frequency and duration. The number of pulses for each pulsed signal was counted manually, using a playback rate of 0.01. In order to ensure a correct quantification of the pulses within the signal of interest, visual inspection of the spectrogram was carried out during the acoustic counting. Repetition rate (clicks/sec) and inter-click interval (ICI) were calculated based on the number of pulses and the duration of each sample.

To illustrate the variation of temporal and spectral features within each signal type the following parameters were obtained for an example of each pulsed signal: bandwidth at 3 dB and 10 dB (mean value and standard deviation based on measurements of each pulse within the signal), variation in repetition rate (difference of values between the first and the last 20% of the signal) and variation in peak frequency (difference of values between the first and the last 20% of the signal).

### Statistical Analyses

Stepwise method of discriminant function analysis (DFA), with Wilks’ Λ [32], was used to identify the acoustic parameters that enable the best discrimination among the selected pulsed signals. For the DFA, we used the groups Slow click trains, Creaks, Squawks and S-BPs, and the acoustic parameters: minimum frequency, peak frequency, duration, and repetition rate. As both repetition rate and ICI result from arithmetic formulas that include the number of pulses and the duration of each pulsed signals, only repetition rate was considered for the DFA analysis.

All the numeric variables were square-root transformed and multivariate outliers were removed. Based on the stepwise DFA, linear discriminant functions were computed. Classification results were used to verify the coherence between the initial labelling of sound types (based on graphical and aural characteristics) and DFA groups. A cross-validation “leave-one-out” method, with 95% confidence intervals, was used for validation.

Additionally, a hierarchical cluster analysis was carried out using the square Euclidean distance and average linkage (within groups) method, for all the pulsed signals rated as fair and good. To determine the number of clusters that best fit the data, the distance values for different stages solution, retrieved from the agglomeration schedule, were evaluated (see [32]). The goal was to assess how many distinct signal types could be determined based on the same acoustic parameters used for the DFA.

One-way ANOVAs (with Welch correction) were performed for the following acoustic parameters: minimum frequency, peak frequency, duration, repetition rate and ICI, using Bonferroni correction (significance level = 0.01). Dunnet’s T3 post hoc tests were performed for pairwise comparisons [32].

All statistical analyses were performed using IBM SPSS Statistics 21 (IBM Inc.)

### Ethics Statement

This study was carried out in strict accordance with the Regulation for Cetacean Observation Activities in Mainland Portugal (Portuguese Law 9/2006). Research in the Sado Estuary was conducted with permission from the Institute for the Conservation of Nature and Forests, Ministry of the Environment (Authorization for Scientific Observation of Cetaceans #2/2011). Acoustic recordings are held by ISPA-IU and Projecto Delfim, and can be made available for use on request.

## Results

**Fig 1:**
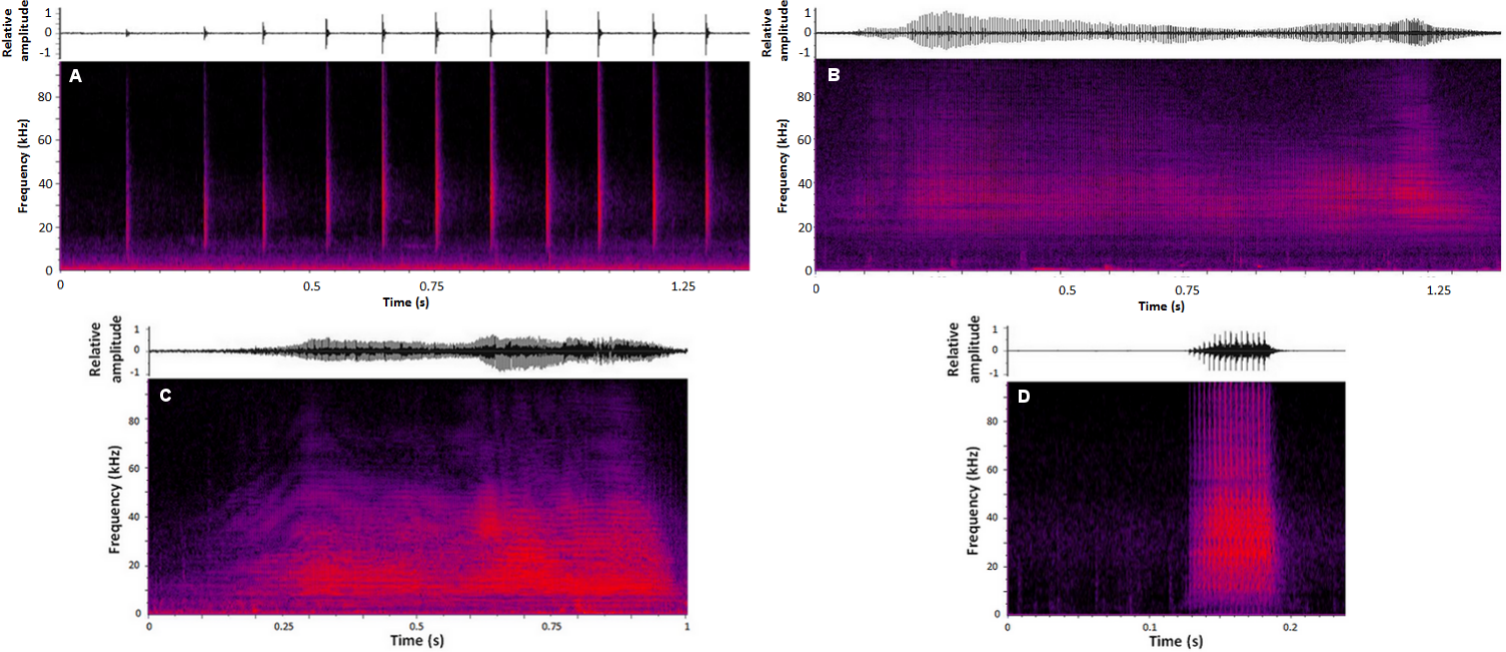
**Examples of pulsed signals produced by bottlenose dolphins in the Sado region, Portugal: (A) Slow click train, (B) Creak, (C) Squawk, (D) S-BP.** The upper panels show sound waveforms, with relative amplitude on the y-axis, and the bottom panels show spectrograms for each signal type, with frequency (kHz) on the y-axis. Time (s) is on the x-axis. Spectrogram settings: FFT 512, Hann window, overlap 50%.

A total of 930 pulsed signals rated as fair or good were initially classified as Slow click trains (N = 393), Creaks (N = 220), Squawks (N = 252) and S-BPs (N = 65), based on aural and graphical characteristics (see Fig 1, S1 Table and S1 Fig).

**Fig 2:**
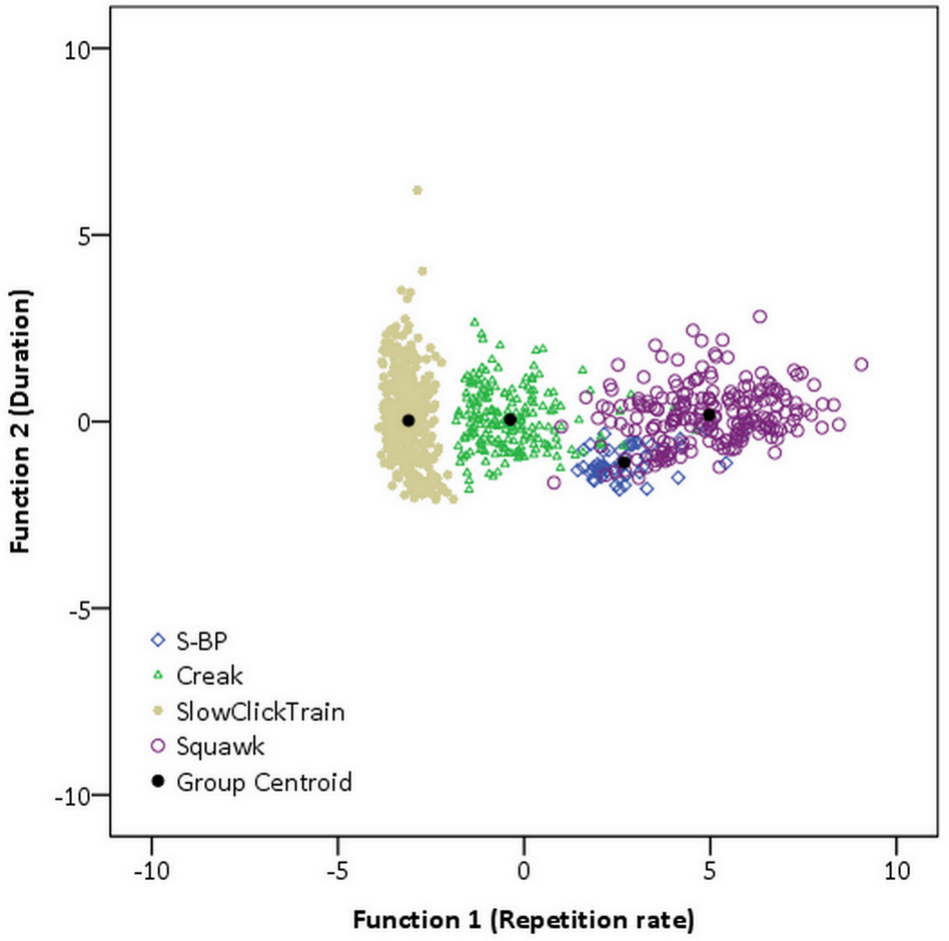
Canonical discriminant analysis plot of the four signal types. Function 1 (Λ = 0.073; χ^2^ (12) = 2174.64; P < 0.001) defined by repetition rate (structure coefficient = 0.98), on the x-axis. Function 2 (Λ = 0.89; χ^2^ (6) = 98.46; P < 0.001) defined by duration (structure coefficient = 0.91), on the y-axis. Functions 1 and 2 represent 99.7% of total variance.

DFA extracted three functions that enable the discrimination of the selected pulsed signals (Fig 2). Function 1 (Λ = 0.073; χ^2^ (12) = 2174.64; P < 0.001) was defined by repetition rate (structure coefficient = 0.98) and accounts for 98.9% of total variance. Function 2 (Λ = 0.89; χ^2^ (6) = 98.46; P < 0.001) was defined by duration (structure coefficient = 0.91), and accounts for 0.8% of variance. Function 3 (Λ = 0.96; χ^2^ (2) = 29.27; P < 0.001) was defined by peak frequency (structure coefficient = 0.95) and minimum frequency (structure coefficient = 0.27), accounting for only 0.3% of total variance.

The stepwise DFA correctly classified 93.5% of the analyzed pulsed signals in their predefined categories. Creaks and Slow click trains were the most consistently allocated to their original categories (93.9% and 98.1%). The few misclassified Slow click trains (N = 7) were initially named Creaks. Misclassified Creaks were initially labelled as S-BP (N = 8) or Squawks (N = 4), but not as Slow click trains. S-BPs and Squawks were also relatively well discriminated (90.7% and 86.1%), with few misclassifications of S-BPs as Squawks (N = 5), and Squawks being labelled as Creaks (N = 2) or Buzzes (N = 28). 91.3% of cross-validated grouped cases were correctly classified.

The output of the hierarchical cluster analysis revealed the existence of two possible cluster solutions (see S2 Fig). For the first solution, two clusters were obtained: cluster 1 comprises all the signals previously labeled as S-BPs, and as Squawks (except for three signals); and cluster 2 combines all the signals labeled as Slow click trains, plus the large majority of signals labeled as Creaks (94%). A second possible solution with five clusters was obtained. In these solution, signals previously labeled as S-BPs, Creaks and Slow click trains were consistently clustered separately: 91% of all S-BPs were grouped in Cluster #1, 84% of Creaks were grouped in Cluster #2 and all Slow click trains were grouped in Cluster #4. Signals previously labeled as Squawks were assigned to three different clusters (#1, #2 and #3).

Different pulsed signals present specific acoustic parameters that differ significantly (Table 1), especially in duration (p<0.001), repetition rate (p<0.001) and ICI (p<0.001).

**Table 1:** Acoustic parameters of pulsed signals produced by bottlenose dolphins in Sado region, Portugal.

	Minimum frequency (kHz)	Peak frequency (kHz)	Duration (sec.)	Repetition rate (clicks/ sec.)	Inter-click-interval (sec.)
**S-BP**	**4.12** ± 3.77^**a**^	**25.97** ± 8.57^**a**^	**0.06** ± 0.04^**a**^^,^^**b**^^,^^**c**^	**287** ± 64^**a**^^,^^**b**^^,^^**c**^	**0.004** ± 0.001^**a**^^,^^**b**^^,^^**c**^
**Creak**	**4.93** ± 3.33^**b**^	**23.73** ± 10.44^**b**^	**1.27** ± 0.93^**a**^^,^^**b**^^,^^**c**^	**83** ± 55^**a**^^,^^**b**^^,^^**c**^	**0. 017** ± 0.010^**a**^^,^^**b**^^,^^**c**^
**Slow click train**	**6.88** ± 3.90^**a**^^,^^**b**^^,^^**c**^	**31.32** ± 5.19^**a**^^,^^**b**^^,^^**c**^	**2.16** ± 1.63^**a**^^,^^**b**^^,^^**c**^	**13** ± 28^**a**^^,^^**b**^^,^^**c**^	**0.128** ± 0.079 ^**a**^^,^^**b**^^,^^**c**^
**Squawk**	**3.94** ± 3.90^**c**^	**21.26** ± 12.18^**c**^	**0.44** ± 0.40 ^**a**^^,^^**b**^^,^^**c**^	**472** ± 161^**a**^^,^^**b**^^,^^**c**^	**0.002** ± 0.001^**a**^^,^^**b**^^,^^**c**^

Values are presented as means ± standard deviation.

^a,b,c^ Significant differences in pairwise comparison, using One-way ANOVAs (with Welch correction) and significance level of 0.01.

The analyses of variance revealed significant differences for all the selected variables among the four classes of pulsed signals: peak frequency (F_Welch (3, 238.84)_ = 69.37, P<0.001), minimum frequency (F_Welch (3, 262.84)_ = 31.48, P<0.001), duration (F_Welch (3, 473.06)_ = 291.77, P<0.001), repetition rate (F_Welch (3, 207.86)_ = 909.29, P<0.001) and inter-click-interval (F_Welch (3, 328.64 = 671.62)_, P<0.001).

Slow click trains were the longest signals in duration followed by Creaks, Squawks and S-BPs. The highest repetition rates and lowest ICI were obtain for Squawks, followed by S-BPs, Creaks and Slow click trains. Minimum and peak frequencies were significantly higher in Slow click trains (p<0.001) but similar for burst-pulses: S-BPs, Creaks and Squawks.

## Discussion

Bottlenose dolphins in the Sado region, Portugal, produce a variety of broadband pulsed signals, comparable to the repertoire of other populations, that have been traditionally classified based on aural characteristics and graphical aspects [30]. Despite the limitations of the human hearing system (essentially its frequency range and temporal processing abilities), our results show a high agreement between traditional classification and quantitative methodologies. As expected, classification based on aural and graphical features performs better with low repetition rate signals (e.g.: slow click trains and creaks), although high percentages of matching classifications were also obtained for Squawks and S-BPs.

A comprehensive classification of pulsed signals is essential for a complete, detailed description of the bottlenose dolphin repertoire and its functional interpretation. The pulsed signals category remains the least understood, both structurally and functionally. All efforts to improve and assess the accuracy of categorizations, and to promote a future universal framework are therefore important.

Pulsed signals produced by bottlenose dolphins in the Sado region range from long, slow click trains to short burst-pulses with very high repetition rate, as it has been described for other delphinid species (e.g.: spinner, spotted and white-beaked dolphins [23, 33], false killer whales [34]). The occurrence of signals with repetition rates that vary along a continuum (e.g., echolocation click trains and burst-pulses emitted with a continuous transition), plus the high variability observed within sound categories, supports the notion of a graded repertoire [35,36]. Graded acoustic repertoires are common in species that live in complex societies, such as canids and non-human primates (e.g.:[37,38]). Furthermore, the level of gradedness of a repertoire has been linked to its potential to encode information [39]. The continuity here observed adds support to the possibility of a dual function for some pulsed signals, i.e., both echolocation and communication (see [40]).

Despite this apparent gradation, our results present statistically significant differences in temporal parameters (duration, repetition rate and ICI) of each type of pulsed signal. Also, the hierarchical cluster analysis clearly divided these signals into coherent clusters, especially Slow click trains, Creaks and S-BPs. Given the dolphins’ hearing system, adapted for both high frequencies and fine-scale time resolution [8], the observed differences in multiple temporal cues may enable the distinction between different vocal units, even if they are produced in continuous sequences. Thus, the existence of discrete pulsed vocal units with specific roles in the bottlenose dolphins’ acoustic repertoire must be considered, even in the sometimes confusing cacophony of graded, pulsed and tonal emissions.

According to the DFA, repetition rate is the feature that best enables the discrimination of different pulsed signals. Repetition rate is also important for the human ability to discriminate sounds. Furthermore, fine-grade temporal discontinuities, on the order of 2–4 ms, are known to be perceived and processed by the human auditory cortex [41,42]. Thus, aural cues have a good potential in perceptual classification even when the ICI is too short for pulse discrimination by the human ear.

In our study, vocal emissions labeled as Slow click trains were markedly distinct signals, with temporal parameters typically reported for echolocation clicks produced by bottlenose dolphins in the wild (e.g.,[15]): long duration (> 1000 ms), slow repetition rate and inter-click-intervals that allow for the two-way transit time. Contrary to other studies [8, 43, 44], Slow click trains had low peak frequency (≈30 kHz). These results may have been caused by the upper frequency limitations of our recording equipment and by the occurrence of both on- and off-axis signals, as already pointed out [45].

Signals labeled as Creaks also appear as distinct vocal units in our DFA, and their spectral characteristics resemble those of terminal buzzes (or creaks) produced by other odontocetes and bats during prey capture efforts that require quick updates of close range scenarios [20,46–50]. Furthermore, creaks are often produced after echolocation clicks as it is described for terminal foraging buzzes [20,46–50] and might have identical function. In our study, Creaks had time-frequency features significantly different from Slow click trains, and the inter-click-intervals, were predominantly lower than the echo-processing lag time reported for this species [8]. However, it is still not clear how dolphins perceive and process these high repetition signals if ICIs are below the limit for auditory temporal resolution [51] and further investigation on this topic is needed. We suggest that an echolocation function should not be excluded; especially since such signals may provide relevant motion related information (such as position shifts and velocity) when targets are at very close ranges.

As for Squawks and S-BPs, these signals fall into the definition of burst-pulses proposed for Hawaiian spinner dolphins and Atlantic spotted dolphins (pulsed signals with ICIs bellow 10 ms) [23], and presented clearly distinct features from echolocation clicks and Creaks. While S-BPs were recorded as sporadic, single, short duration emissions, Squawks were abundant vocalizations recorded both as isolated calls, and following Creaks emissions.

Burst-pulses of short durations have been previously documented for captive and wild bottlenose dolphins [13,52]. Although such signals have been described as similar in duration to the S-BPs we present, their repetition rates (≈ 500 pps) and peak frequency (≈20 kHz) are closer to those of Squawks in our study. Furthermore, the reported burst-pulses are common vocalizations, often emitted in sequences, unlike the signals labelled by us as S-BPs. Due to the singular characteristics of S-BPs and their pattern of emission, we suggest that these burst-pulses emitted in the Sado estuary may be a new/unreported signal in the acoustic repertoire of this species.

Very high repetition rate signals (>500 ppm) such as Squawks have been documented in different social contexts (both affiliative and agonistic) and hypothesized to function as indicators of the animals’ physical and emotional state during interactions [13,22,24]. Recently, burst-pulses with spectral characteristics similar to Squawks, recorded in a continuum with echolocation click trains and creaks have been described as “an acoustic signal of food reward expectation” [53]. Synchronized squawks that resemble isolated Squawks have been reported during agonistic interactions [54]. In our study, Squawks had high variability in their time-frequency features and were recorded with different patterns of emission. Thus, it is possible that the category Squawks combines several variants in a range of signals with different contextual use.

Interestingly, the cluster analysis results, especially solution 1, validates a distinction between the signal types that can be linked with biosonar tasks (Slow click trains and Creaks) and other pulsed signals that probably have a communication function (Squawks and S-BPs).

## Conclusions

Pulsed signals are a conspicuous component of bottlenose dolphins’ repertoire and evidence supports their important role both in foraging/feeding events and intraspecific communication. Defining more correct and natural sound categories and sub-categories is indispensable to the description of a species’ acoustic repertoire. We highlight the potential of combining graphical aspects of spectrograms and quantitative analysis in the process of pulsed-sounds classification.

Our results document significant differences in time-frequency characteristics of pulsed signals produced by bottlenose dolphins. These findings point to the existence of a complex repertoire of pulsed vocalizations.

Future studies should examine the patterns and contexts of production of each specific signal type, as it is likely that these emissions are determined by specific social and environmental factors.

## Supporting Information

S1 Fig Example of pulsed signals emitted in a sequence (Slow click train–Creak–Squawk). Upper panel shows the signal waveform, with relative amplitude on the y-axis. Bottom panel shows the spectrogram, with frequency (kHz) on the y-axis and time (s) is on the x-axis. Spectrogram settings: FFT 512, Hamming window, overlap 50%.
(TIF)

S2 Fig Dendrogram of the hierarchical cluster analysis using square Euclidean distance and average linkage (within groups) method. Cluster analysis was performed using square-root transformed variables (minimum and peak frequency, repetition rate and duration) of different pulsed signals produced by bottlenose dolphins in Sado region, Portugal. Two solutions with two and five clusters, respectively, are presented at X-axis with red lines. Solution 1—Cluster #A: comprises all the signals previously labeled as S-BPs, and as Squawks (except three samples); Cluster #B: combines all the signals labeled as Slow click trains, plus the large majority of signals labeled as Creaks (94%). Solution 2—Cluster #1: includes the majority of S-BPs (91% of all emissions, N = 54), plus Squawks (N = 60) and Creaks (N = 10); Cluster #2: mainly composed by Squawks (N = 92 samples), plus S-BPs (N = 3) and Creaks (N = 2); Cluster #3: includes only fast repetition rate signals—squawks (N = 61) and S-BPs (N = 3); Cluster #4: comprises only Slow click trains (N = 369); Cluster #5: includes the majority of Creaks (N = 183) and three Squawks (N = 3). Y-axis represents the rescaled distance cluster combine, with a red line intercepting the cut-off value for the proposed cluster solution, based on the agglomeration schedule.
(TIF)

S1 Table Temporal and spectral features within each signal type (illustrative example). (PDF)
