# Supplementary figures and images for: A Quantitative Analysis of Pulsed Signals Emitted by Wild Bottlenose Dolphins
Source: PLoS One. 2016 Jul 6;11(7):e0157781. doi: 10.1371/journal.pone.0157781 (PMC4934784; doi:10.1371/journal.pone.0157781)

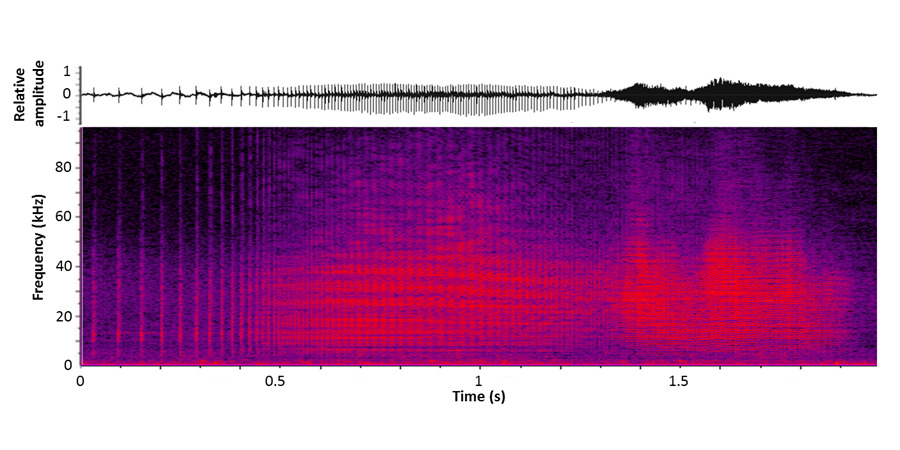

Supplement: S1 Fig — Upper panel shows the signal waveform, with relative amplitude on the y-axis. Bottom panel shows the spectrogram, with frequency (kHz) on the y-axis and time (s) is on the x-axis. Spectrogram settings: FFT 512, Hamming window, overlap 50%. (TIF) [file pone.0157781.s001.tif]

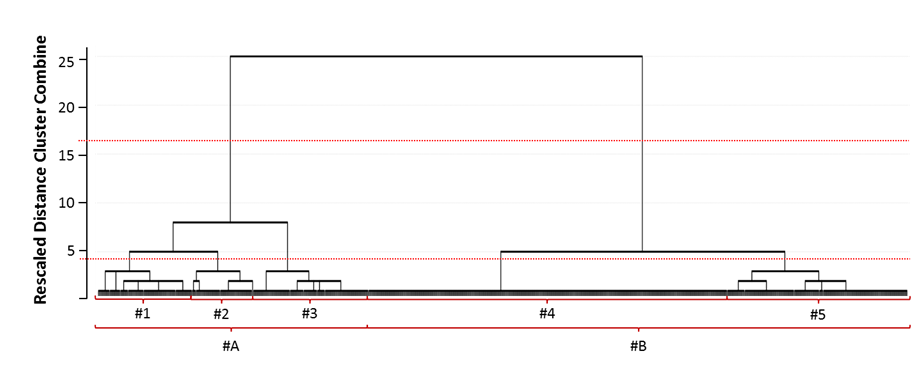

Supplement: S2 Fig — Cluster analysis was performed using square-root transformed variables (minimum and peak frequency, repetition rate and duration) of different pulsed signals produced by bottlenose dolphins in Sado region, Portugal. Two solutions with two and five clusters, respectively, are presented at X-axis with red lines. Solution 1—Cluster #A: comprises all the signals previously labeled as S-BPs, and as Squawks (except three samples); Cluster #B: combines all the signals labeled as Slow click trains, plus the large majority of signals labeled as Creaks (94%). Solution 2—Cluster #1: includes the majority of S-BPs (91% of all emissions, N = 54), plus Squawks (N = 60) and Creaks (N = 10); Cluster #2: mainly composed by Squawks (N = 92 samples), plus S-BPs (N = 3) and Creaks (N = 2); Cluster #3: includes only fast repetition rate signals—squawks (N = 61) and S-BPs (N = 3); Cluster #4: comprises only Slow click trains (N = 369); Cluster #5: includes the majority of Creaks (N = 183) and three Squawks (N = 3). Y-axis represents the rescaled distance cluster combine, with a red line intercepting the cut-off value for the proposed cluster solution, based on the agglomeration schedule. (TIF) [file pone.0157781.s002.tif]
