# Supplementary material for: A Quantitative Analysis of Pulsed Signals Emitted by Wild Bottlenose Dolphins
Source: PLoS One. 2016 Jul 6;11(7):e0157781. doi: 10.1371/journal.pone.0157781 (PMC4934784; doi:10.1371/journal.pone.0157781)
Supplement: S1 Table — (PDF) [file pone.0157781.s003.pdf]

S1 Table. Temporal and spectral features within each signal type (illustrative example).

	**Bandwidth at 3 dB (kHz)**	**Bandwidth at 10 dB (kHz)**	**Variation in repetition rate (clicks/sec.)**	**Variation in peak frequency (kHz)**
**S-BP**	13.45 ± 4.46	41.92 ± 8.67	0	3.75
**Slow click train**	15.39 ± 4.79	28.29 ± 8.75	- 1.20	- 9.00
**Creak**	11.91 ± 6.22	33.55 ± 9.62	55.76	0.66
**Squawk**	9.56 ± 6.66	30.00 ± 7.15	73.20	0.66
